# Self‐Aggregation of Convective Clouds With Interactive Sea Surface Temperature

**DOI:** 10.1029/2020MS002164

**Published:** 2020-11-03

**Authors:** S. Shamekh, C. Muller, J.‐P. Duvel, F. D'Andrea

**Affiliations:** ^1^ Laboratoire de Météorologie Dynamique IPSL, École Normale Supérieure, PSL Research University, CNRS Paris France

**Keywords:** self‐aggregation, sea surface temperature, cloud‐resloving simulation, convection

## Abstract

This study investigates the feedbacks between an interactive sea surface temperature (SST) and the self‐aggregation of deep convective clouds, using a cloud‐resolving model in nonrotating radiative‐convective equilibrium. The ocean is modeled as one layer slab with a temporally fixed mean but spatially varying temperature. We find that the interactive SST decelerates the aggregation and that the deceleration is larger with a shallower slab, consistent with earlier studies. The surface temperature anomaly in dry regions is positive at first, thus opposing the diverging shallow circulation known to favor self‐aggregation, consistent with the slower aggregation. But surprisingly, the driest columns then have a negative SST anomaly, thus strengthening the diverging shallow circulation and favoring aggregation. This diverging circulation out of dry regions is found to be well correlated with the aggregation speed. It can be linked to a positive surface pressure anomaly (PSFC), itself the consequence of SST anomalies and boundary layer radiative cooling. The latter cools and dries the boundary layer, thus increasing PSFC anomalies through virtual effects and hydrostasy. Sensitivity experiments confirm the key role played by boundary layer radiative cooling in determining PSFC anomalies in dry regions, and thus the shallow diverging circulation and the aggregation speed.

## Key Points

- An interactive sea surface temperature (SST) has a deceleration‐acceleration impact on aggregation depending on SST response to drying
- The deceleration‐acceleration is due to first warming (enhanced shortwave) and second cooling (enhanced longwave) of surface in dry regions
- A high‐pressure anomaly that forms in dry regions drives a shallow circulation that explains the speed of aggregation

## 1 Introduction

The spontaneous organization of deep convective clouds into a single cluster, which is known as self‐aggregation, has been found across a wide range of 2‐D and 3‐D cloud‐resolving models (CRMs) and global climate models (GCMs) with different configurations and domain shapes and sizes (Held et al., 1993; Muller & Held, 2012; Nakajima & Matsuno, 1988; Wing, 2019; Wing et al., 2017). Self‐aggregation typically starts with the appearance and growth of persistent dry areas devoid of deep convection. The growth of those dry regions leads to the confinement of convection to a remaining small fraction of the simulation domain. This phenomenon results in a reduction of domain‐averaged water vapor content, and consequently a significant enhancement of outgoing longwave radiation to space. If relevant to the real world, this self‐aggregation of deep convective clouds could potentially impact the climate sensitivity (Coppin & Bony, 2018; Mauritsen & Stevens, 2015).

Several studies have investigated the physical processes responsible for this phenomenon. Most of these studies use fixed sea surface temperature (SST). In a seminal study, Bretherton et al. (2005) point out the importance of surface fluxes and atmospheric radiative cooling for self‐aggregation. They find that enhanced radiative cooling in the lower troposphere in dry regions leads to the formation of a shallow flux from dry to moist regions. This shallow circulation transports moist static energy (MSE) from dry to moist regions, that is, up‐gradient (from low‐MSE to high‐MSE regions), thus reinforcing the MSE gradient and the aggregation of convection.

The importance of atmospheric radiative cooling has been confirmed by other studies as well; however, based on model and simulation configuration, different radiative feedbacks can drive the self‐aggregation. Muller and Held (2012) show that the low‐level clouds longwave radiation is necessary for self‐aggregation to occur from homogeneous initial conditions, while clear‐sky and high‐cloud longwave radiative feedbacks are sufficient to maintain the aggregation. Yang (2018) mentions the importance boundary layer moisture variance and boundary layer differential radiative cooling. Wing and Emanuel (2014) also highlight the importance of clear‐sky free‐tropospheric radiative cooling. Other studies mention the importance of free‐tropospheric moisture and convection‐moisture feedback (Craig & Mack, 2013; Tompkins & Craig, 1998), surface fluxes feedbacks (Coppin & Bony, 2015), and also cold pool‐convection feedback (Jeevanjee & Romps, 2013). In summary, several physical processes contribute to the onset and maintenance of convective self‐aggregation. It is still unclear which one of those feedbacks, if any, dominates. In particular, more work is desirable to clarify the role of low‐level versus free‐tropospheric radiative feedbacks on driving self‐aggregation.

One robust feature, though, is the significant increase of MSE variance with self‐aggregation (Wing & Emanuel, 2014). As mentioned above, the radiatively driven shallow circulation and concomitant up‐gradient MSE transport are believed to play a key role. Indeed, the shallow circulation transports low‐level air with high MSE to already moist regions. A strong shallow flux can result in a strong up‐gradient transport of MSE, thus negative gross moist stability, which is known to favor aggregation (Bretherton et al., 2005). The importance of boundary‐layer differential radiative cooling rates, between dry and moist regions, in driving this shallow circulation has been suggested by Muller and Bony (2015). Using a conceptual, analytical model of the boundary layer, Naumann et al. (2017) and Naumann et al. (2019) further investigate the divergent shallow circulation out of a dry region driven by enhanced boundary layer radiative cooling, and how it compares to the shallow circulation driven by SST anomalies. Their dry theoretical model confirms that a shallow circulation can be maintained for differences in radiative boundary‐layer cooling rates larger than 1 K day^−1^. The circulation strength is comparable to that caused by SST differences of a few Kelvins (Naumann et al., 2017), or even larger when moisture effects are accounted for (Naumann et al., 2019). The circulation follows from colder boundary layer temperatures and thus increased hydrostatic surface pressures, in regions with larger boundary layer radiative cooling.

Worth to mention that all the studies mentioned so far used fixed homogeneous surface temperature. Using a nonhomogeneous SST (constant in time but inhomogeneous in space) or an interactive SST (evolving in time) can also change the occurrence of self‐aggregation and the dominating feedback (Hohenegger & Stevens, 2016; Liu & Moncrieff, 2008; Müller & Hohenegger, 2020; Shamekh et al., 2019; Tompkins, 2001). Introducing an SST anomaly can dictate the preferred location of convection (Tompkins, 2001) and thus impact the self‐aggregation. When a circular SST anomaly (constant in time) is imposed, the aggregation process is significantly accelerated (Shamekh et al., 2019), due to the large‐scale circulation that develops in response to the stronger upward mass flux over the warm region. Consistently, Back and Bretherton (2009) show that a boundary layer divergent flow forms in response to an SST gradient, which can re‐enforce deep convection.

A few studies have also investigated the response of self‐aggregation to an interactive SST. In that case, the SST evolves in space and time according to the local energy budget (see section 2.3 for more details). When SST is allowed to interact with the atmosphere, the self‐aggregation is typically delayed or prevented (Coppin & Bony, 2017; Grabowski, 2006; Hohenegger & Stevens, 2016). Using a GCM coupled with a slab ocean, Grabowski (2006) shows that warm SST anomalies form under the cloud free area by enhanced shortwave radiation, which reaches the surface. In the GCM, convective clusters follow the warm SST anomaly and result in an easterly propagating convective cluster similar to the Madden Julian oscillation (Arnold & Randall, 2015). In a similar set‐up, Coppin and Bony (2017) find that the convective aggregates prefer to stay on the maximum of SST gradient, which also results in the similar propagation found by Grabowski (2006).

Using a CRM and a domain of (576 km)^2^, Hohenegger and Stevens (2016) investigate the impact of different slab depths on the aggregation of convection. They find that the coupling between the SST and the atmosphere delays the onset of self‐aggregation or prevents it completely if the slab is very shallow (1 m). They suggest that this delay is the result of the formation of an SST gradient, which opposes the boundary layer divergent flow (shallow circulation mentioned above) known to be important for the development of convective aggregation, a hypothesis that we further investigate and quantify here. Of particular interest are the following questions:

- What is the impact of interactive SST on aggregation, and how do surface variables evolve as the aggregation progresses?
- Do SST anomalies oppose the shallow circulation between dry and moist regions?
- What is the relative importance of the shallow versus the deep circulation in the MSE transport and time scale of aggregation?

The next section describes the CRM and simulations in more detail, as well as the index used to quantify the convective aggregation. Section 3 describes the impact of interactive SST on aggregation for various slab depths and mean SST and describes in detail the evolution of surface properties in one simulation. Section  4 investigates the physical processes behind the sensitivity to mixed layer depth and domain‐mean SST, notably the relative roles of boundary layer radiative cooling anomalies and SST anomalies in setting up a shallow circulation. Concluding remarks are offered in section 5.

## 2 Method

### 2.1 Cloud‐Resolving Model

The CRM used is the model System for Atmospheric Modeling (SAM) Version 6.11.1 (Khairoutdinov & Randall, 2003). This model solves the anelastic equations of conservation of momentum, water (with six species present in the model, water vapor, cloud liquid, cloud ice, precipitating rain, precipitating snow, and precipitating graupel), and energy. The relevant energy for moist convection is the MSE, as it is conserved (approximately, i.e., neglecting viscous and subgrid‐scale effects) under adiabatic processes including the phase change of water. More precisely in this model, the so‐called “frozen” MSE is conserved during moist adiabatic processes, including the freezing of precipitation. The frozen MSE is given by

(1) $$MSE=c_{p}T+gz+L_{v}q-L_{f}q_{ice},$$

with the specific heat capacity of air at constant pressure *c*_*p*_, temperature *T*, gravity *g*, height *z*, latent heat of evaporation *L*_*v*_, water vapor specific humidity *q*_*v*_, latent heat of fusion *L*_*f*_, and specific humidity of all ice phase condensates *q*_*ice*_.

The subgrid‐scale turbulence is modeled using a Smagorinsky‐type parameterization, and we use the one‐moment microphysics formulation, following Bretherton et al. (2005) and Muller and Held (2012). Surface fluxes are computed using bulk formulae. Further information about the model can be found in Khairoutdinov and Randall (2003).

All simulations use interactive radiation, using the radiation code from the National Center for Atmospheric Research (NCAR) Community Atmosphere Model Version 3 (CAM3; Collins et al., 2006). For simplicity, we neglect the diurnal cycle and use the daily mean incoming solar insolation of 413 W m^−2^ (same setting as  Tompkins & Craig, 1998).

### 2.2 Experimental Setup

The model domain is square, doubly periodic in both horizontal directions *x* and *y*. We run simulations with horizontal domain size (576 km)^2^. The horizontal resolution is 3 km, and the vertical grid spacing increases gradually with height, with the first level at 25 m and a resolution of 50 m close to the sea surface, reaching a vertical resolution of 500 m in the midtroposphere. There are 64 vertical levels, which span 27 km in the vertical. This includes a sponge layer in the upper third of the domain (from *z* = 18 km to 27 km) where the wind is relaxed to zero in order to reduce gravity wave reflection and buildup. No large‐scale forcing or wind is imposed in the domain. We neglect the Earth's rotation, a reasonable approximation in the tropics where the Coriolis parameter is small.

The initial conditions for the different domain‐averaged SSTs are obtained from smaller domain runs with the corresponding SST at radiative‐convective equilibrium (RCE) ([96 km]^2^ run to 50 days), then using time and domain‐averaged profiles of the last 5 days. We run simulations with three different depths of slab: *H* = 5, 10, and 50 m, at two domain‐averaged SSTs = 300 and 305 K. This allows us to explore the impact of interactive SST on aggregation and compare the sensitivity to slab depth to the impact of changing domain‐averaged SST by 5 K. We also perform fixed SST simulations for both SST = 300 and 305. Note that fixed SST is mathematically equivalent to infinite slab depth; thus, the results should converge to the fixed SST simulation when *H* increases. A simulation will be referred to by its depth of slab and its SST so that, for example, Simulation H5SST305 has slab depth of 5 m and SST = 305 K.

As the time to equilibrium is longer with interactive SST, and thus the computation is more expensive, in particular with shallow slab depth, we stop the simulations when the metric used for the aggregation progress (introduced below in section 2.4) reaches its maximum and drops back down to its equilibrium value. Worth to mention that after this drop, the metric oscillates around a value between 0.4 and 0.5 and does not depend on slab depth or mean SST.

### 2.3 Slab Ocean

One technical complication with tropical simulations using interactive SST is that the incoming solar radiation in the tropics exceeds the threshold for a runaway greenhouse gas warming (Pierrehumbert, 2010). In the tropics, oceanic and atmospheric transport of energy out of the tropics compensates the energy imbalance at the top of the atmosphere and prevents excessive warming. This is not the case in simulations of an isolated tropical region with periodic boundary conditions that lacks the transport of energy out of the tropics by the Hadley cell and mean ocean circulation, as well as eddy transport.

Several solutions have been proposed to overcome this issue, for example, reducing the incoming solar flux (Cronin et al., 2015), adding a constant deep ocean flux (Romps, 2011), or relaxing the domain average SST to a target temperature (Semie & Tompkins, 2016), that is, adding a deep ocean flux that ensures little drift in domain‐mean SST.

Aggregation is known to be sensitive to the domain‐mean SST (e.g., Wing & Emanuel, 2014). Thus, in order to separate the effects of domain‐mean SST and of spatial inhomogeneities, here we follow Semie and Tompkins (2016) and relax the domain‐averaged ocean mixed layer temperature $\bar{SST}$ toward a fixed target temperature *SST*_0_ (see Appendix A for a brief discussion of simulations with a constant deep ocean flux and their domain mean SST drifts). This relaxation method allows us to keep the domain‐averaged SST constant over time while it allows the SST to vary locally according to the evolution equation:

(2) $$\rho_{w}c_{p,w}H\left(\frac{dSST}{dt}+\frac{\bar{SST}-SST_{0}}{\tau_{0}}\right)=Q_{SW}^{N}+Q_{LW}^{N}+LHF+SHF,$$

where *ρ*_*w*_ denotes water density, *c*_*p*,*w*_ is the specific heat capacity of water at constant pressure, *H* the depth of the slab, and *τ*_0_ the relaxation time scale, which is constant and equal to 2 hr in all of our simulations (this value was empirically determined to avoid significant drift in the domain mean SST). All the terms on the right hand side of Equation 2 are positive downward (increase SST) and negative upward (decrease SST). *LHF* and *SHF* denote surface latent and sensible heat fluxes (up to a minus sign), and $Q_{SW}^{N}$ and $Q_{LW}^{N}$ stand, respectively, for shortwave and longwave net radiative flux at the surface, with

(3) $$Q_{LW}^{N}=LW_{d}-\sigma SST^{4},$$

where *σ* is the Stephen‐Boltzmann constant and *LW*_*d*_ the downward longwave flux at the surface.

Also,

(4) $$Q_{SW}^{N}=(1-\alpha )SW_{d}=(1-\alpha )SW_{TOA}e^{\left(-\tau /\mu_{0}\right)},$$

where *α* is albedo, *SW*_*d*_ is downward shortwave flux at the surface, *μ*_0_ is a constant that depends on the zenith angle, *τ* is the shortwave optical depth, and *SW*_*TOA*_ is the incoming shortwave flux at the top of the atmosphere. In our simulations, *τ* changes only by changes in water vapor and cloud water content of the atmosphere, as the other ingredients do not change.

Using Equation 2, we can find an equation for spatial SST anomalies $SST^{\prime }=SST-\bar{SST}$:

(5) $$\frac{dSST^{\prime }}{dt}=\frac{1}{\rho_{w}c_{p,w}H}\left(Q_{SW}^{N^{\;\prime }}+Q_{LW}^{N^{\;\prime }}+LHF^{\prime }+SHF^{\prime }\right).$$

Worth to mention that the relaxation term disappears as it is the same everywhere in the domain. Thus, the spatial variability of SST arises from spatial variations of the energy flux at the surface.

### 2.4 Analysis Framework

To follow the progress of aggregation, we use column relative humidity *CRH* (Shamekh et al., 2019; Wing & Cronin, 2016).

(6) $$CRH=\frac{\int q_{v}\;\rho \;dz}{\int q_{v,sat}\;\rho \;dz},$$

where *q*_*v*,*sat*_ is the saturation water vapor specific humidity, *ρ* is the air density, and the vertical integration is done over the troposphere. We use CRH for our analysis, as it is less dependent on surface temperature compared to the integrated column water vapor (PW); thus, it allows us to compare the aggregation progress at different SSTs.

In our simulations, deep convection does not occur in regions with *CRH* < 0.6; thus, we define the dry patch as the area with *CRH* < 0.6. With the progress of aggregation, these dry patches grow and merge so that they take a larger and larger fraction of the domain. As the growth of the dry patches is the main feature in all our simulations, to follow the progress of aggregation, we use the fractional area covered by dry patches, which we will refer to as the aggregation index.

## 3 The Impact of Interactive SST on the Aggregation of Convective Clouds

In this section, we first provide an overview of the main results regarding the impact of interactive SST on the progress of self‐aggregation in our simulations. We then study in detail one simulation with slab depth = 5 m and domain mean SST = 305 K (hereafter H5SST305), as processes are found to be qualitatively similar in all simulations. Notably, we investigate in detail how interactive surface temperatures affect the surface pressure anomaly in dry regions, and thus the shallow circulation between dry and moist regions known to play an important role in the aggregation process.

### 3.1 Overview of Results

**Figure 1:**
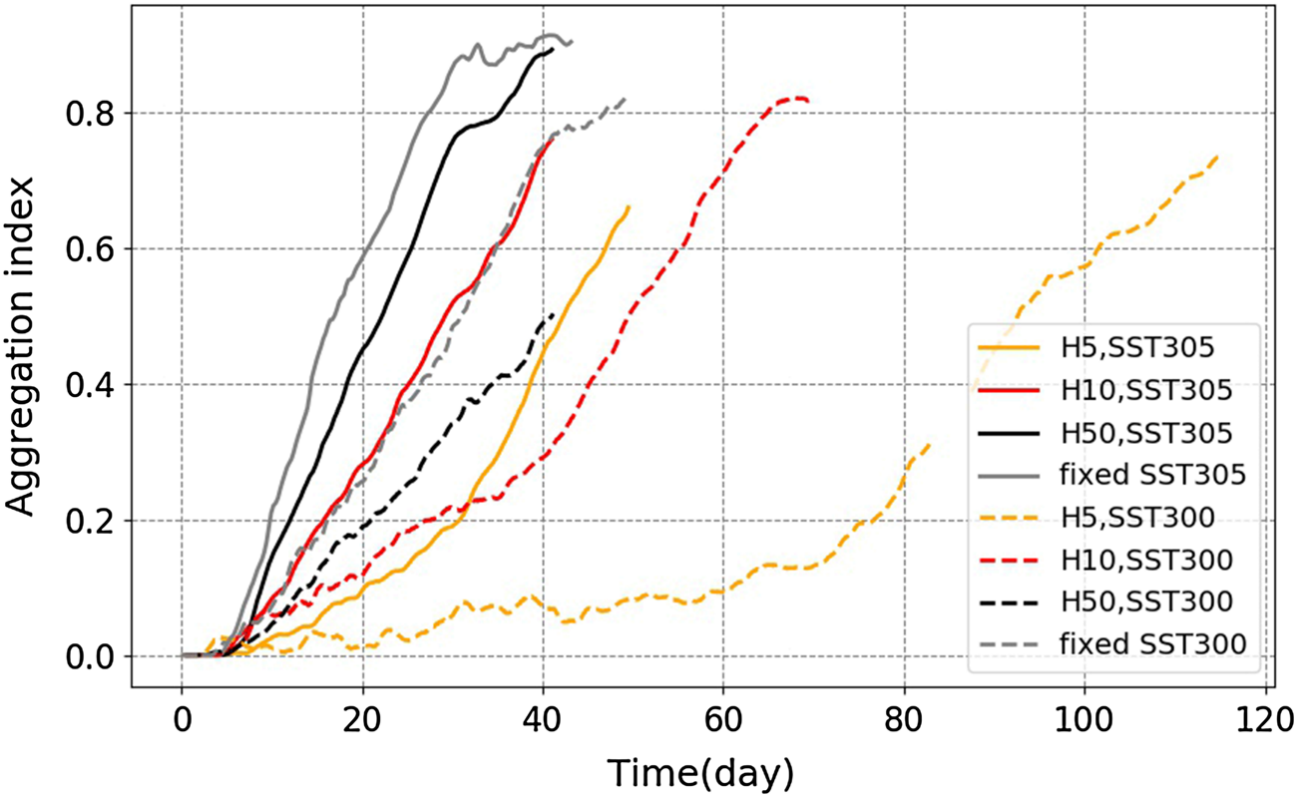
Time series of the aggregation index (area _*CRH* < 0.6_) for *SST*_0_ = 300 K (dashed lines) and *SST*_0_ = 305 K (plain lines) and different slab depths. Simulations with fixed SST are also shown for reference (gray lines). (We note in passing that the few days missing in the H5SST300 simulation, around Day 85, are due to a technical issue but do not affect the results discussed here.)

Figure 1 shows the aggregation index for the simulations with interactive SST with different slab depths and with SSTs 300 and 305 K. To compare the timing of self‐aggregation in our simulations, we simply compare the time at which the aggregation index reaches its maximum (Figure 1).

Generally speaking, introducing a slab ocean with interactive surface temperature delays self‐aggregation (Figure 1). Consistently, the fixed SST simulations (which correspond to infinite slab depth) are faster at both 300 and 305 K than the interactive SST simulations with the same mean SST. This is consistent with previous studies on the impact of slab ocean on self‐aggregation (Bretherton et al., 2005; Hohenegger & Stevens, 2016). Here, with the slab depths that we examined, the aggregation always proceeds, but it is significantly delayed with a shallow slab as, for example, H10SST305 and H5SST305 delay the self‐aggregation by 12 and 25 days, respectively, compared to fixed SST simulation (Figure 1). The delays obtained with interactive SST (tens of days) are comparable to the delays obtained when reducing the SST. Indeed, similar tens of days delays are found when decreasing the SST from 305 to 300 K for a given slab depth (Figure 1).

Note that in some of the simulations, there is a period of slower increase of the aggregation index (e.g., H5SST300 before Day 70 or H5SST305 before Day 30) before the index starts its faster monotonic increase. We refer to this delayed period, for which the aggregation index is not increasing significantly, as latency.

We now investigate in more detail surface properties in one of the simulations since, as mentioned earlier, properties are found to be qualitatively robust in all the runs. Of particular interest are the evolution of surface temperature and surface pressure in dry regions, and how these impact the circulation.

### 3.2 SST Anomalies

**Figure 2:**
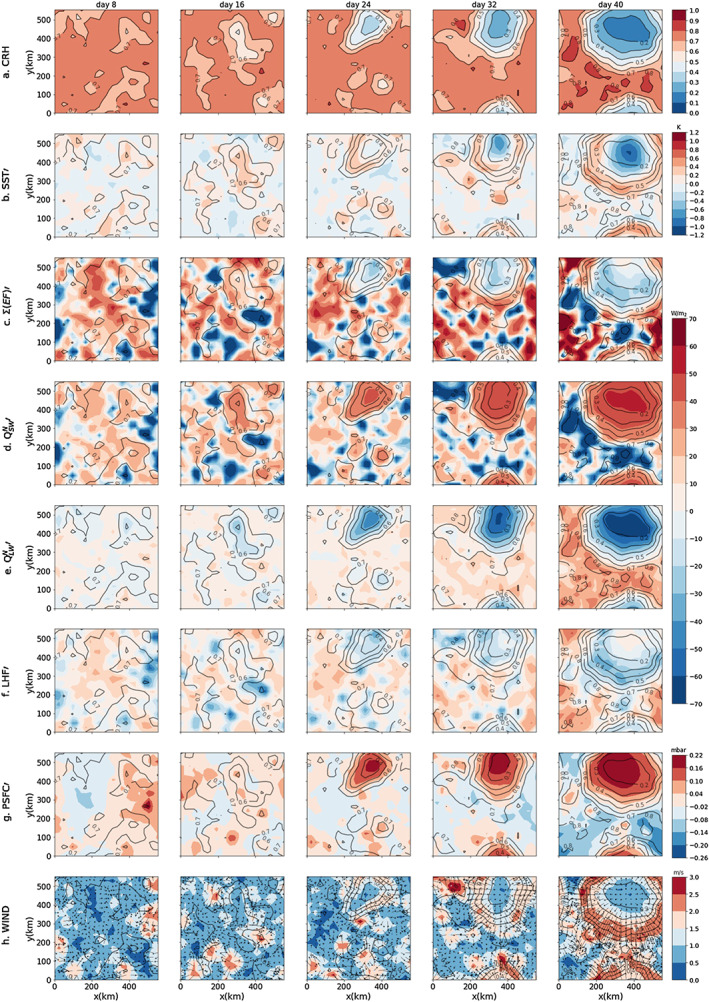
The rows show daily mean of (a) CRH, (b) SST anomaly, (c) net energy flux at the surface, (d) net shortwave radiative flux anomaly at the surface, (e) net longwave radiative flux anomaly at the surface, (f) surface latent heat flux anomaly, (g) surface pressure anomaly, and (h) surface wind (color) with arrows showing the direction of the wind. Columns show the time progress of each variable. The data are taken from Simulation H5SST305 averaged over 6 hr of the day mentioned on the top of each column. Each panel is further smoother by 16*16 grids column averaging. The contours of CRH are repeated in all panels to ease comparison. In all flux plots (c–f) downward (upward) flux is shown with positive (negative) sign.

**Figure 3:**
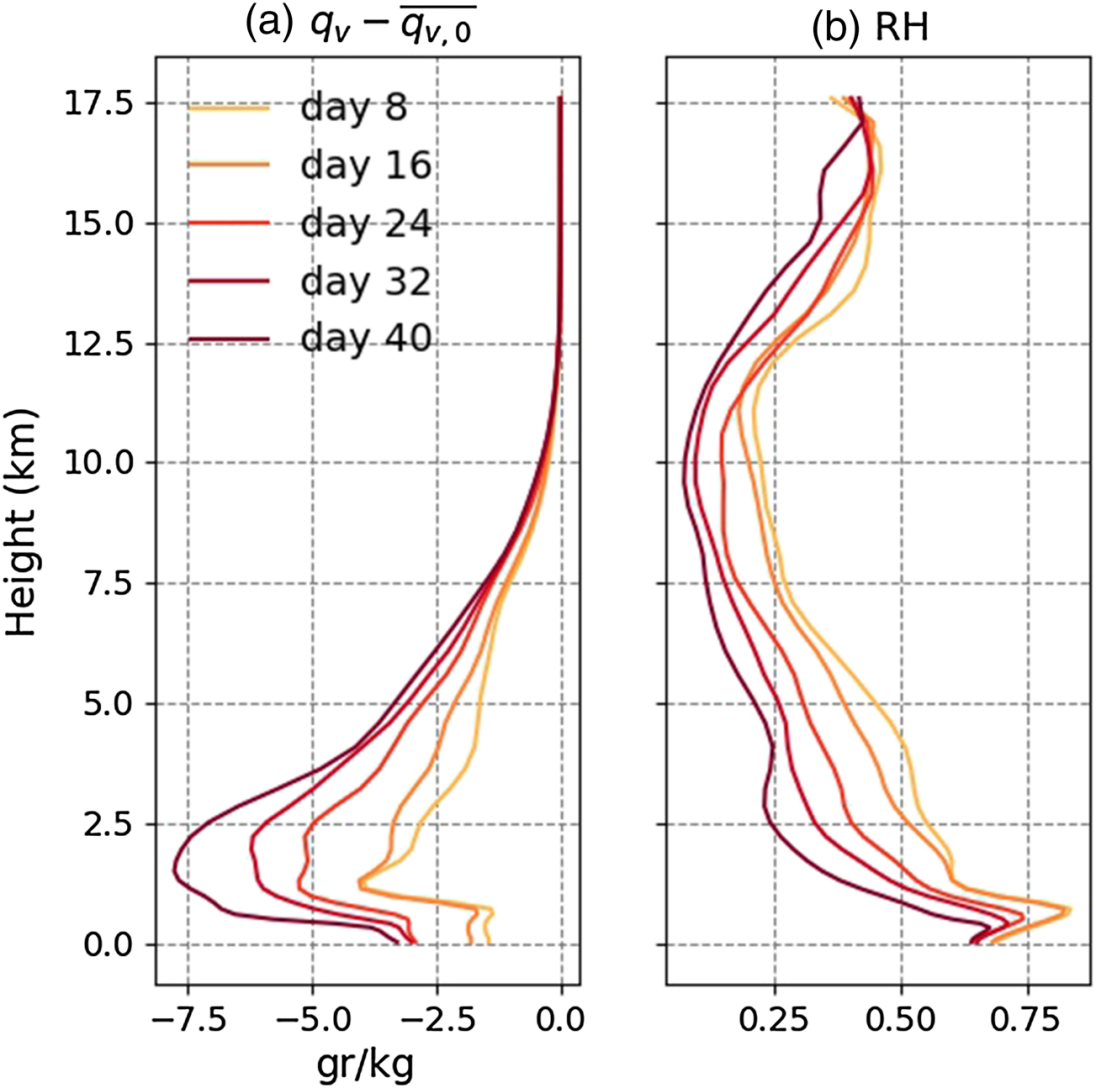
The vertical profiles of (a) total change in specific humidity ( $qv-\bar{q_{v,0}}$) and (b) relative humidity averaged over the dry patch (defined by *CRH* < 0.6) for Simulation H5SST305.

Figure 2 shows the time evolution of several variables, including SST anomaly and the surface energy budget anomaly (right‐hand side of Equation 5) in Simulation H5SST305. The first dry patches are well detectable at Day 16 (Figure 2a). These dry patches grow where they first appear without significant displacement. By Day 40, a single circular dry patch exists and covers half of the domain. It is worth to mention that the dryness is more pronounced in the free troposphere, specially at early time, and reaches the boundary layer by Day 24 (Figure 3).

We see that the SST anomalies exhibit two different stages of evolution: (1) an early stage warming, which happens when the dry patch is newly formed and still has large amount of column water vapor, so that shortwave warming dominates, and (2) a later stage cooling at the center of the dry patch surrounded by a ring of warm water, which is located at the edge of the dry patch. This pattern appears with further dryness of the dry patch, so that longwave cooling dominates at the center of the dry patch; it is worth to mention that the dry patches become cloud free from early time (see Figure 2d). In the following, we investigate each of these stages separately.

#### 3.2.1 Early Stage Warming

The temperature anomaly underneath the dry patches at their early appearance (up to Day 16) is positive; thus, the first impact of a dry patch on the surface temperature is warming (Figure 2b). This early‐stage warming in dry regions can be understood by looking at the net energy flux anomaly (hereafter ΣEF′) at the surface (Equation 5 and Figure 2c). In dry regions, ΣEF′ is initially positive, thus leading to an SST increase with time. ΣEF′ is predominantly determined by shortwave and longwave radiative fluxes at the surface, with also a small contribution by surface latent heat flux. The sensible heat flux is very small thus negligible.

The early‐stage warming is mainly because of an enhancement in the shortwave radiative flux at the surface (SWNS), as the dry patches become cloud free and more transparent from the very beginning, letting larger amounts of shortwave radiation reach the surface and building up positive SST anomalies locally (Figures  2b and 2d). These cloud‐free dry patches experience a reduction in total column water vapor (Figure 3). As noted above, the dryness starts from the free troposphere and is not significant in the boundary layer (up to Day 16).

When the free troposphere becomes dry, the boundary layer and the surface can radiatively cool more efficiently in the longwave (Emanuel et al., 2014). The enhancement of net longwave radiative flux at the surface (LWNS) is clear in Figure 2e; however, as the dry patches still have a large amount of water vapor, especially in the boundary layer, the surface cooling by LWNS is smaller than warming by enhanced shortwave radiative flux.

All together, at early stage of dry patches, surface warming by SWNS is more efficient compared to cooling by LWNS (and LHF) leading to the formation of warm SST anomalies.

#### 3.2.2 Later Stage Cooling

By Day 24, with further dryness and expansion of the dry patches, surprisingly, the surface at the center of one of the dry patches with lowest CRH becomes colder than the area around it (Figure 2b; the dry patch at *x* = 400 km and *y* = 500 km) so that a cold anomaly surrounded by a ring of warm water forms. CRH over the ring of warm water is large (roughly comparable with CRH in the dry patches at Day 16); thus, its warming is caused by the dominance of SWNS versus LWNS and LHF. The cooling at the center of the dry patch indicates that further drying of the dry patches can have a cooling effect on the surface temperature underneath them by increasing LWNS with an additional albeit small contribution from enhanced LHF. The surface latent heat fluxes (Figure 2f) increases as a result of increased gradient of specific humidity between the surface and the first layer of the atmosphere, as the dryness has already reached the boundary layer (Figure 3), further enhanced by surface winds in the ring around the cold anomaly (Figure 2h). The enhanced LWNS is the result of low amount of column water vapor that results in a smaller downward longwave radiative flux at the surface and allows the LWNS to increase. This enhancement is well seen in Figure 2e, Day 24 at *x* = 400 km and *y* = 500 km.

To summarize, at the center of the dry patch, around Day 24, cooling by LWNS dominates (and LHF) results in a negative trend in surface temperature anomaly, while at the edge of the dry patch, shortwave warming overcomes longwave cooling (and the small contribution from latent heat flux) so that a ring of warm water forms. This pattern of warm ring‐cold center further intensifies with dryness of dry patch. By Day 40, LHF at the center of the dry patch reduces due to the reduction of SST and small surface wind. At this stage, the cold patch persists as LWNS remains larger than SWNS and LHF.

After Day 40, the reduction in LHF results in an increase in surface temperature anomaly at the center of the dry patch (not shown). The warmer center in return increases LHF, so that the SST anomaly and LHF at the center of the dry patch oscillate slowly around an equilibrium value (not shown).

These SST anomalies can potentially affect the aggregation speed by impacting the surface pressure anomaly in dry regions, an aspect that we further investigate in the following section.

### 3.3 Surface Pressure Anomaly

At the early stage of the simulation, the surface pressure anomaly (hereafter PSFC) is slightly positive under some of the dry patches (Figure 2g, Day 16). A similar high‐pressure anomaly in dry region has been found by Yang (2018). A positive PSFC anomaly builds up a divergent flow at the surface, which exports low‐level moist air from the dry to the moist regions (Figure 2h). This flow further dries the dry regions, which strengthen and expand. The divergent flow then increases the horizontal variance of water vapor, which is correlated with the progress of aggregation. As mentioned in section 1, this low‐level divergent circulation and the concomitant up‐gradient MSE transport are well documented in aggregation simulations (Bretherton et al., 2005; Muller & Bony, 2015; Muller & Held, 2012; Yang, 2018). Here we further investigate the origin of this high surface pressure anomaly in dry regions, and its link with specific humidity, radiation, and SST anomaly.

**Figure 4:**
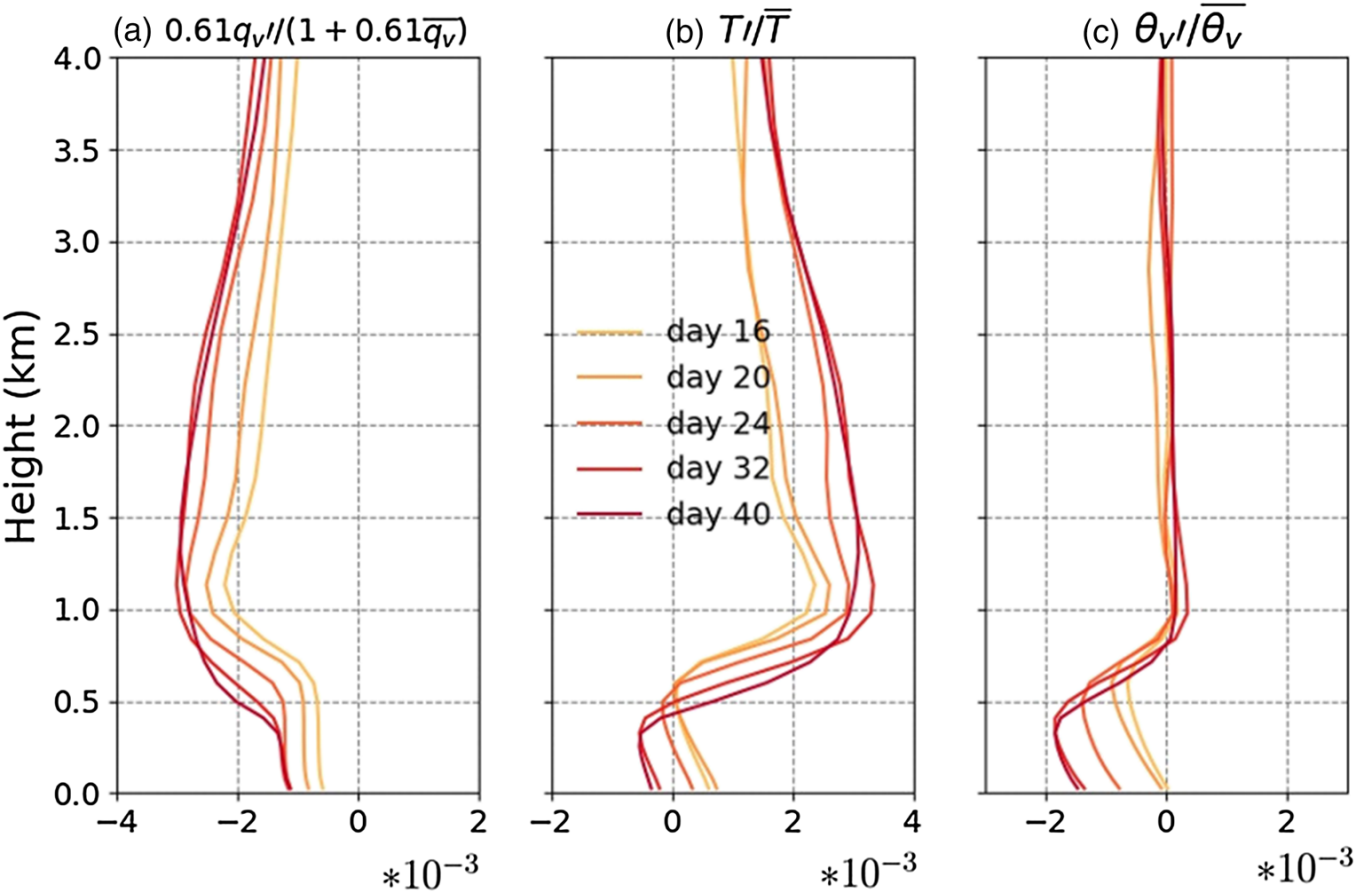
Plots show the contribution of (a) qv anomalies and (b) temperature anomalies into (c) virtual potential temperature anomalies. Each line is 1 day averaged over the dry patch (*CRH* < 0.6) for Simulation H5SST305.

**Figure 5:**
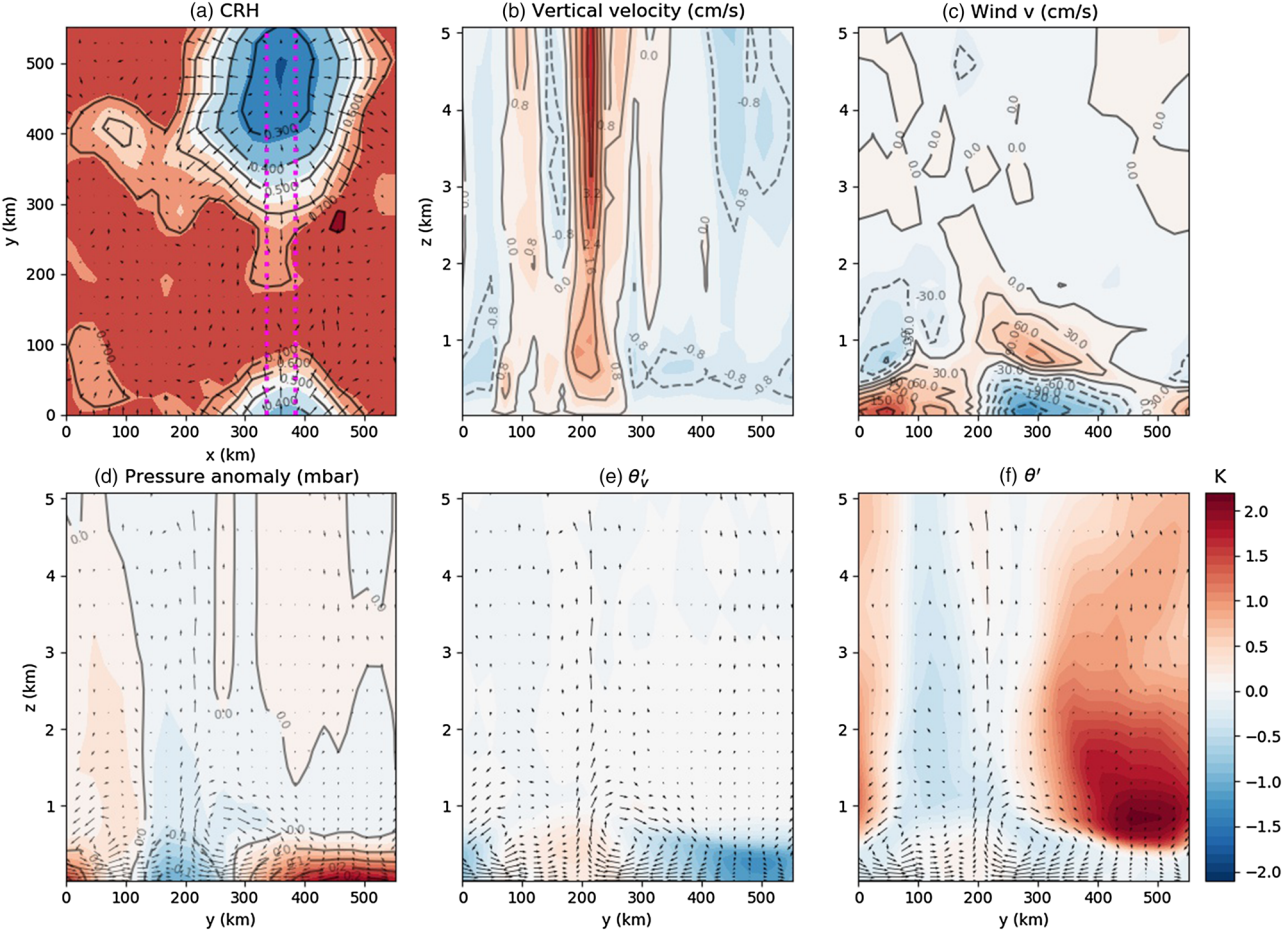
Simulation H5SST305 (a) shows the top view of CRH at Day 32. (b–e) The vertical profile between magenta line in panel (a) averaged in *x* direction. The dry patch is centered around *y* = 500 km. (b and c) The vertical profile of vertical and horizontal velocity, respectively. (d–f) Pressure anomaly (mbar), *θ*_*v*_ anomaly, and *θ* anomaly, respectively. Colorbar corresponds to panels (e) and (f).

**Figure 6:**
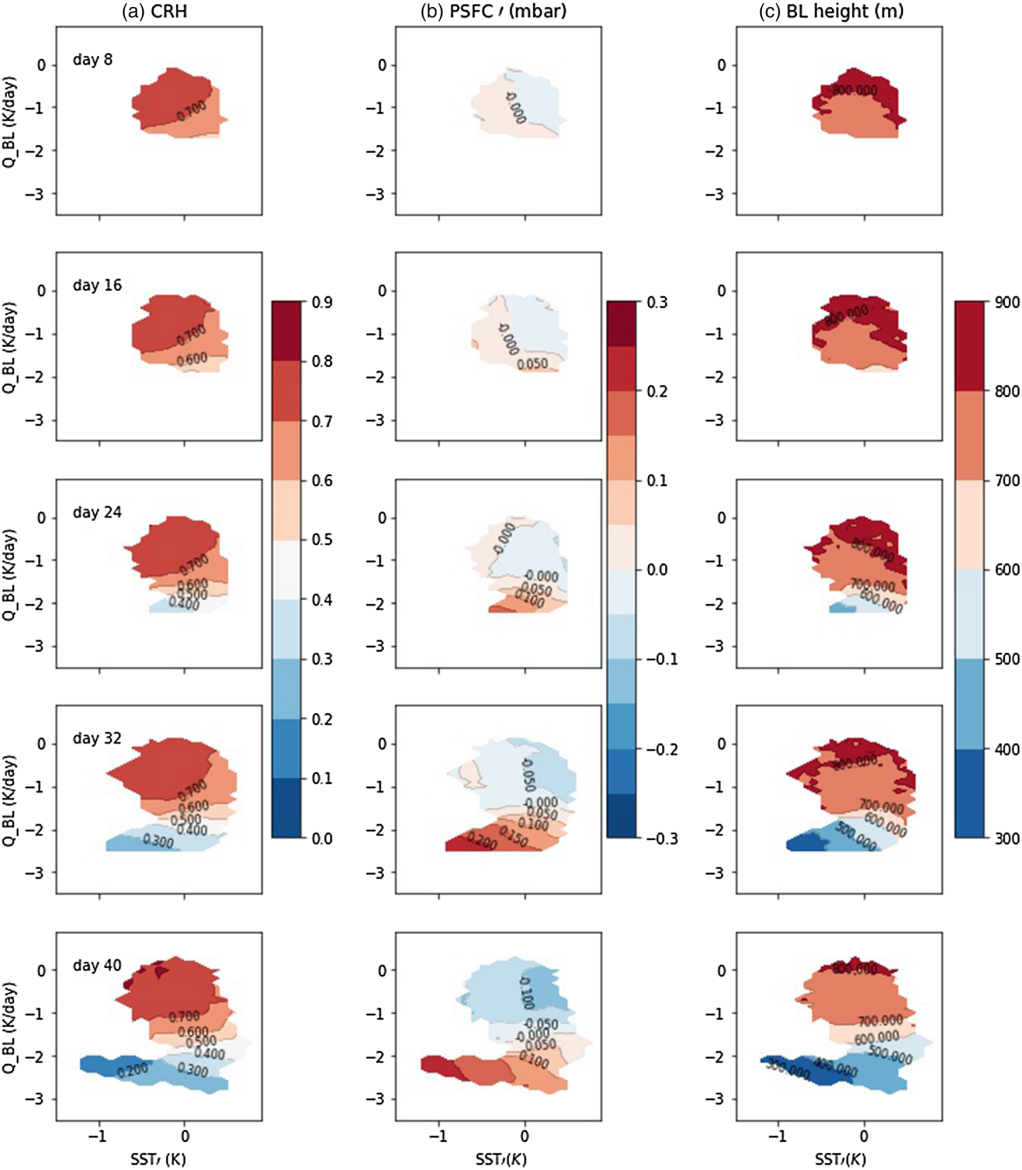
CRH (column a), PSFC anomaly (column b), and boundary layer height (column c) for Simulation H5SST305. The *x* axis is SST anomaly, and the *y* axis is radiative cooling averaged over the boundary layer *Q*_*BL*_. To compute the top of the boundary layer, we use the *q*_*v*_ profile and determine the first altitude above ground where it has a maximum curvature.

The horizontal gradients of virtual temperature are small in the free troposphere (Figures 4 and 5d) consistent with the weak temperature gradients of tropical regions where the Coriolis parameter is small (Sobel et al., 2001). Thus, the surface pressure anomaly in dry regions is related to the boundary layer density anomaly. To determine the depth of the boundary layer for each grid, we find the height at which the gradient of specific humidity has its largest change. Using this definition, we see that the boundary layer height is uniform at the beginning of the simulation when moisture is uniformly distributed. But with the progress of aggregation and further dryness of dry regions, the boundary layer height reduces significantly in dry regions compared to the moist region. However, we did not find the change in boundary layer height to impact the surface pressure anomaly (Figure 6c). Thus, in the following discussion on the source of the surface pressure anomaly, we neglect the impact of boundary layer height.

As mentioned above, the high PSFC anomaly in dry region is generated due to density anomaly in the boundary layer. To compare the air density, we use virtual potential temperature (hereafter *θ*_*v*_): Moistening/warming the air reduces the air density thus increases *θ*_*v*_. Assuming hydrostatic balance in dry region (where we do not have any convection or even shallow clouds), the surface pressure anomaly depends on the virtual temperature anomaly, itself a function of temperature and moisture anomalies. In fact, we interpret the surface pressure anomaly in the dry regions as being the consequence of SST anomalies and boundary layer radiative cooling anomalies there. This is motivated by earlier work showing the key role played by low tropospheric radiation in self‐aggregation (Muller & Bony, 2015), and by the theoretical model of Naumann et al. (2019, 2017) showing the similar or even larger radiatively driven shallow circulation compared to that driven by SST gradients. A locally warmer SST tends to warm and moisten (through enhanced surface latent heat flux) the column and oppose the positive PSFC anomaly. On the other hand, a locally enhanced boundary layer radiative cooling (hereafter *Q*_*BL*_, negative for a cooling) can cool and dry the boundary layer through subsidence and generate a positive PSFC anomaly. In Appendix B1, we confirm the key role played by locally enhanced boundary layer radiative cooling in sensitivity simulations: Homogenizing it prevents the aggregation in our simulations, while homogenizing radiation in the free troposphere has little impact. Yang (2018) argued that locally enhanced boundary layer radiative cooling has a negative feedback on the aggregation before and at the very early time of dry patch appearance and then turning to a positive feedback when the dry patches start growing. Here we argue that the boundary layer radiative cooling is necessary and creates a positive feedback from the beginning of dry patches appearance through its impact on the surface pressure anomaly. The difference between our results and Yang (2018) can be related to the fact that his feedback analysis (through the budget of vertically integrated available potential energy) only includes the direct diabatic effect of radiation but does not take into account the indirect effect of the radiatively induced circulation and associated moisture transport.

From the early stage of the dry patch (Day 16), the boundary layer moisture anomaly is negative and creates a negative *θ*_*v*_ anomaly (Figure 4a); in other words, a positive pressure anomaly. The existence of a negative moisture anomaly and its positive contribution to positive PSFC anomaly in dry region is consistent with the finding of Yang (2018). The temperature anomaly in dry patch is slightly positive at the beginning but then becomes negative with further progress of the dry patch (Figure 4b). So, up to Day 24, the temperature profile of the boundary layer opposes the formation of a positive PSFC anomaly, but after turning negative, it favors the positive PSFC anomaly, consistent with the SST anomaly discussed in the previous section, first positive opposing and second negative favoring aggregation. This opposition‐acceleration effect can be seen in Figure 1, orange solid line for which the index shows two stages: from the start of simulation up to Day 30 or so for which the aggregation index increases slowly followed by an accelerated stage when the SST in the dry region becomes more and more negative.

Figure 6 shows the evolution of PSFC in *Q*_*BL*_ and *SST′* space. At Day 16, on the lower part of the panel (i.e., at more negative *Q*_*BL*_), SST anomaly is positive thus warming the column while *Q*_*BL*_ is large and cooling the column. Figure 4b shows that the temperature anomaly of the dry region is still opposing the positive PSFC anomaly. But this opposition has been reduced by boundary layer radiative cooling. Indeed, the cooling generates subsidence (not shown), yielding a drying and concomitant moisture effect on *θ*_*v*_, which dominates the temperature effects and lead to the formation of a positive PSFC anomaly (Figure 4a).

As Figure 4 shows, the opposite effects of *q*_*v*_ and T on *θ*_*v*_, up to Day 24 or so, might explain why some of the dry patches do not persist at early stage and disappear. This opposing impact results in a small *θ*_*v*_ anomaly thus small PSFC anomaly that has a low chance of persistence. By Day 24, the center of the dry patch has a cold SST anomaly (section 3.2); furthermore, *Q*_*BL*_ is enhanced as the column is drier (Figure 6). Thus, the temperature of boundary layer also favors positive PSFC anomaly, so that PSFC anomaly shows a significant enhancement (Figures 2g and 6).

The high pressure in dry region results in divergence and further expansion of dry patches. The dry patches are cloud free, even in the early warm stage. More precisely, convection stays outside the warm water, and there is not any significant enhancement of convective activity at the edge of the dry patches with warm surface (Supporting Information S1). The studies on the impact of SST gradient on convective activities (e.g., Liu & Moncrieff, 2008) find an enhancement of rainfall where SST gradient is larger. This is due to a convergent flow induced by SST gradient. In our simulations, the regions of warm SST anomalies form in response to the atmosphere drying and are divergent; thus, no convection or shallow cloud forms. In equilibrium state, when aggregation is fully reached, the surface under the convective area becomes cold; thus, the surface is divided into three regions: a cold and very dry, a warm and dry, and a moist and cold convective region. The moist convective region though seems not to move toward the warm anomaly and stays confined and motionless, for tens of days after reaching equilibrium. Note that in GCM studies of aggregation with interactive SST (Coppin & Bony, 2017; Grabowski, 2006), the moist patch always follows the warm SST anomaly, which forms under the dry patch: a “cat and mouse” dynamics. The time scale of this propagation is on the order of hundreds of days. To verify whether this propagation occurs in our simulations, a long run lasting at least a year would be required.

The picture that emerges is that enhanced boundary layer radiative cooling in dry regions dries the boundary layer and thus through virtual effects creates a high‐pressure anomaly there. This positive PSFC anomaly is partially offset by warmer SSTs at early stages of the aggregation process. Once the dry patch is dry enough, the SST anomaly reverses because of enhanced surface cooling by longwave radiation and the colder SST adds to the boundary layer cooling in dry regions, and by hydrostasy to positive PSFC anomaly. The sign and magnitude of PSFC anomaly have a large impact on the persistence and growth of dry patches. More precisely, a positive PSFC anomaly ensures the expansion of dry patches by exporting MSE via a boundary layer divergent flow out of dry patches. As this divergent flow is found to be crucial for the speed of aggregation, we explore it in more detail in the following section.

### 3.4 Divergent Flow

The surface wind is divergent in dry patches from their early stage (Figure 2h). From earlier studies on aggregation, it is known that this low‐level divergent circulation is key in transporting moisture and MSE out of dry patches, strengthening moisture and MSE gradients. The dry patches then expand (e.g., Bretherton et al., 2005). Consistent with the theoretical model of Naumann et al. (2017), we saw that this low‐level circulation can be related to the persistence of a high surface pressure anomaly, itself related to negative moisture anomaly, stronger boundary layer cooling, and SST anomaly. Here we investigate further the vertical structure of this circulation. Figure 5 shows a vertical cross section of winds, pressure, potential temperature (*θ*), and virtual potential temperature (*θ*_*v*_) anomalies at Day 32. We see that the divergent flow is indeed located in the boundary layer (below 1 km or so; see Figure 5c) where *θ*_*v*_ has a large variance (Figure 5e). In the free troposphere, consistent with theoretical expectations in the tropics (Sobel et al., 2001), *θ*_*v*_ anomalies are small.

**Figure 7:**
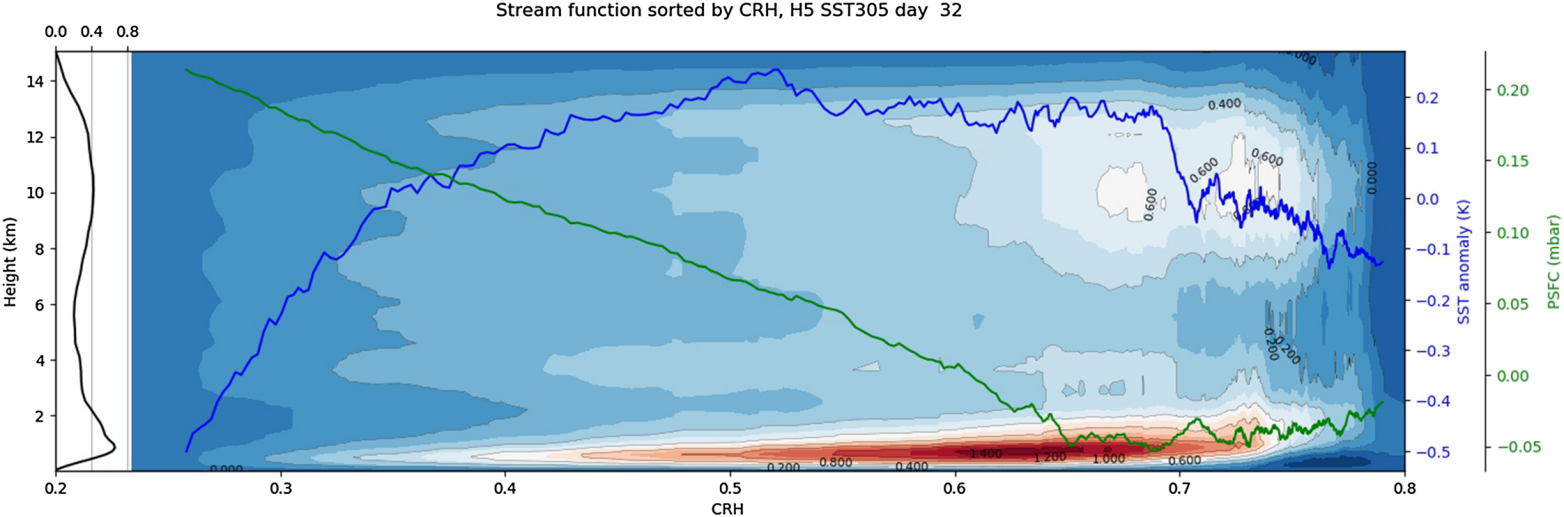
Stream function for Simulation H5SST305 at Day 32 (color and contours, kg m^−2^ s^−1^). Black line on the left side of the panel shows the stream function averaged over the domain. Blue line shows the SST anomaly, and green line shows PSFC anomaly both sorted by CRH.

The divergent flow can also be shown using the stream function (Ψ) in CRH—height space (Bretherton et al., 2005):

(7) $$\Psi_{i}(z)=\Psi_{i-1}(z)+\underset{CRH\in [CRH_{i-1},CRH_{i}]}{\sum }\bar{\rho (z)}w(z),$$

where *i* is the index of CRH bin (sorted), *w* is the vertical velocity summed in the CRH bin *i*, $\bar{\rho }$ is the domain‐mean density profile, and Ψ_0_ = 0 for all *z*. This stream function represents the total mass transport between low‐ and high‐CRH bins. Figure 7 shows that it has one maximum below 3 km (which we refer to as the boundary layer divergence) and one maximum in the free troposphere (which we refer to as the deep circulation). The boundary layer divergence extends from dry to moist regions where the PSFC gradient is maximum, consistent with the boundary layer divergence of the snapshot shown in Figure 5.

Indeed, the maximum of the stream function Ψ at low levels is exactly equal to the boundary layer divergence out of dry regions and into moist regions:

(8) $$\Psi_{max}=\int_{0}^{z_{max}}\rho u_{max}\;dz,$$

where *z*_*max*_ denotes the height of the stream function maximum and *ρu*_*max*_ is the total mass transport through the CRH bin of the stream function maximum (about 0.65 on Figure 7), that is, total mass transport from low CRH to high CRH values.

As Figures 5 and 7 show, part of the boundary layer divergence returns to the dry region higher in the boundary layer (around ≈2 km), while the rest of it is transported upward. Here we define the shallow circulation as part of the flux that stays in the boundary layer (circulation below 4 km or so). It is given by Ψ_*max*_ − Ψ_*min*_, where Ψ_*min*_ is the minimum of the stream function (around 4 km). It is this shallow circulation that exports low‐level air with high MSE from dry regions and imports air with low MSE at the top of the boundary layer into dry regions, that is, up‐gradient. This shallow circulation thus has negative gross moist stability, leading to aggregation, for example, Neelin and Held (1987), Bretherton et al. (2005), and Raymond et al. (2009).

The deep circulation, on the other hand (which is given by Ψ_*max*,*deep*_ secondary stream function maximum around 10 km), disfavors aggregation by transporting air with high MSE (found at high altitudes, above 10 km on Figure 7) out of the moist convective region, thus down‐gradient (positive gross moist stability). Therefore, the more bottom heavy the circulation, the more favored the aggregation. We further explore the role of this shallow circulation, and the importance of the ratio of shallow to deep circulation on the speed of aggregation, in the following section, where we extend our study to various slab ocean depths and mean SSTs.

## 4 The Impact of Slab Depth and SST on Self‐Aggregation

### 4.1 Delayed Aggregation With Shallow Mixed Layer and Cold SST

To investigate the impact of slab depth and domain mean SST on our findings, we extend the analysis of section 3 to two more depths of the slab, *H* = 10 and 50 m, and one more domain mean surface temperature *SST*_0_ = 300 K. As was shown earlier (Figure 1 and section 3.1), introducing an interactive SST typically slows down the aggregation, and the delay obtained (tens of days) is comparable to the delay due to decreasing the mean SST in fixed SST simulations (from 305 to 300 K). Based on the aggregation index (Figure 1), we define two regimes: Regime 1 when the aggregation index increases slowly (we refer to this period as *latency*) and Regime 2 with monotonic and fast increase of the aggregation index to its maximum value (we refer to this period as *transient*). In the following, we elaborate on the impact of slab depth and domain mean SST on each of these periods.

#### 4.1.1 Latency

**Figure 8:**
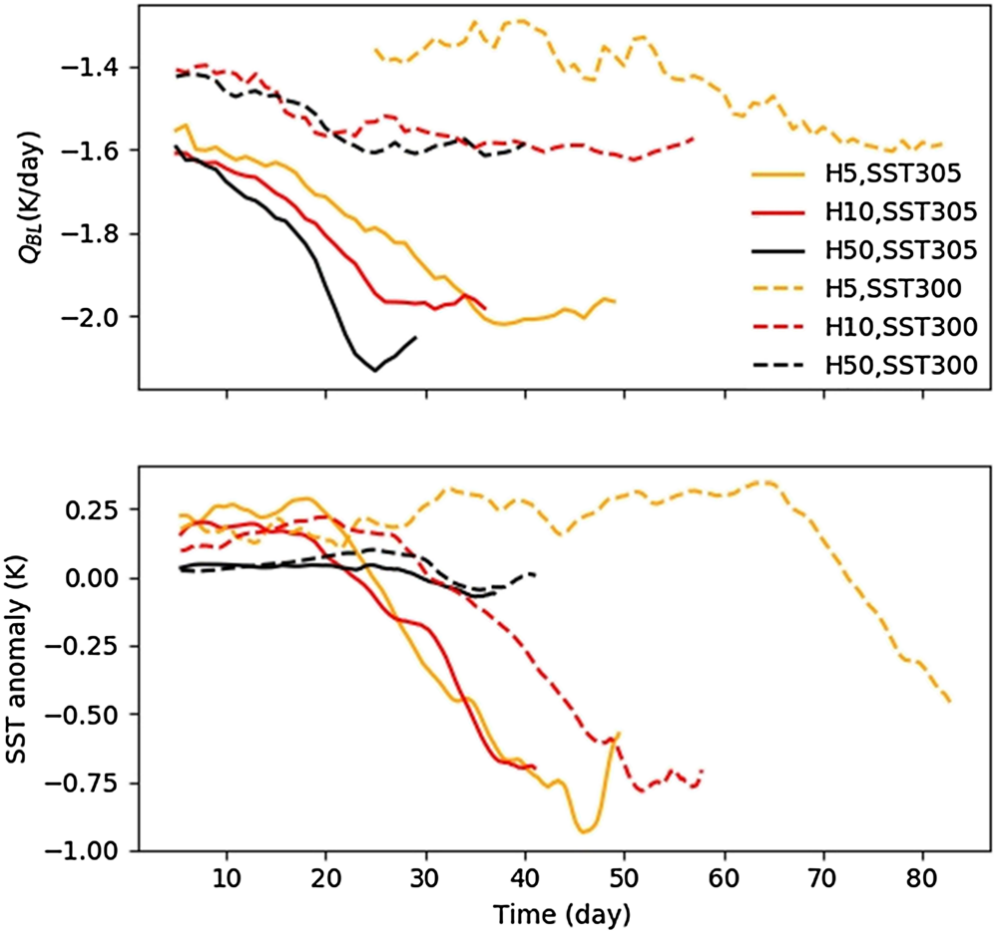
Time series of (top) boundary layer radiative cooling anomaly (difference between dry and moist region) and (bottom) surface temperature anomaly averaged over the dry patch.

Figure 8 shows the relative contributions of SST anomaly and boundary layer cooling in dry regions in the different simulations. We see that with a shallower mixed layer depth, the warm SST anomaly in dry regions is larger and persists longer than with a deeper mixed layer. Thus, with a shallower slab, the probability that a dry patch, at its first stage, recovers its moisture and disappears is larger.

This process can significantly delay the aggregation or potentially prevent it if the warm anomalies are large enough. Otherwise, if the negative feedback by SST is not as strong as positive feedback by radiative cooling, the aggregation index increases but slowly. For example, the aggregation indices in Simulations H5SST300 and H10SST300 have, respectively, the latency equal to 70 and 35 days. Thus, the larger the warm SST anomalies (or the shallower the slab), the longer the latency. As Figure 1 shows, when the slab depth is large (especially at 305 K), the latency goes to zero. As expected, the deepest slab is close to simulations with fixed SST.

We also find that for a given slab depth, the latency is longer at 300 K compared to 305 K. Specifically, the latency for H5SST305 is about 30 days while for H5SST300 it is around 70 days. We interpret this longer latency as being the result of the significantly weaker *Q*_*BL*_ at 300 K (Figure 8), while SST anomalies have similar magnitudes. Weaker *Q*_*BL*_ has a smaller contribution to the high‐pressure PSFC, so the negative feedback from SST anomaly becomes more important, leading to a longer latency.

The reason why *Q*_*BL*_ is smaller at 300 K is not clear; however, it could be related to the specific humidity of the free troposphere. At 305 K, the free tropospheric specific humidity is larger (due to the thermodynamic constraint given by the Clausius‐Clapeyron equation, which predicts an approximately exponential increase of specific humidity with temperature for constant relative humidity). Consequently, the decrease of specific humidity due to subsidence in dry regions, which is proportional to the specific humidity, is more rapid at 305 K compared to 300 K. The free tropospheric dryness allows the boundary layer to radiatively cool more efficiently and have a stronger contribution to PSFC. We will come back to this large‐scale circulation in section 4.2.

**Figure 9:**
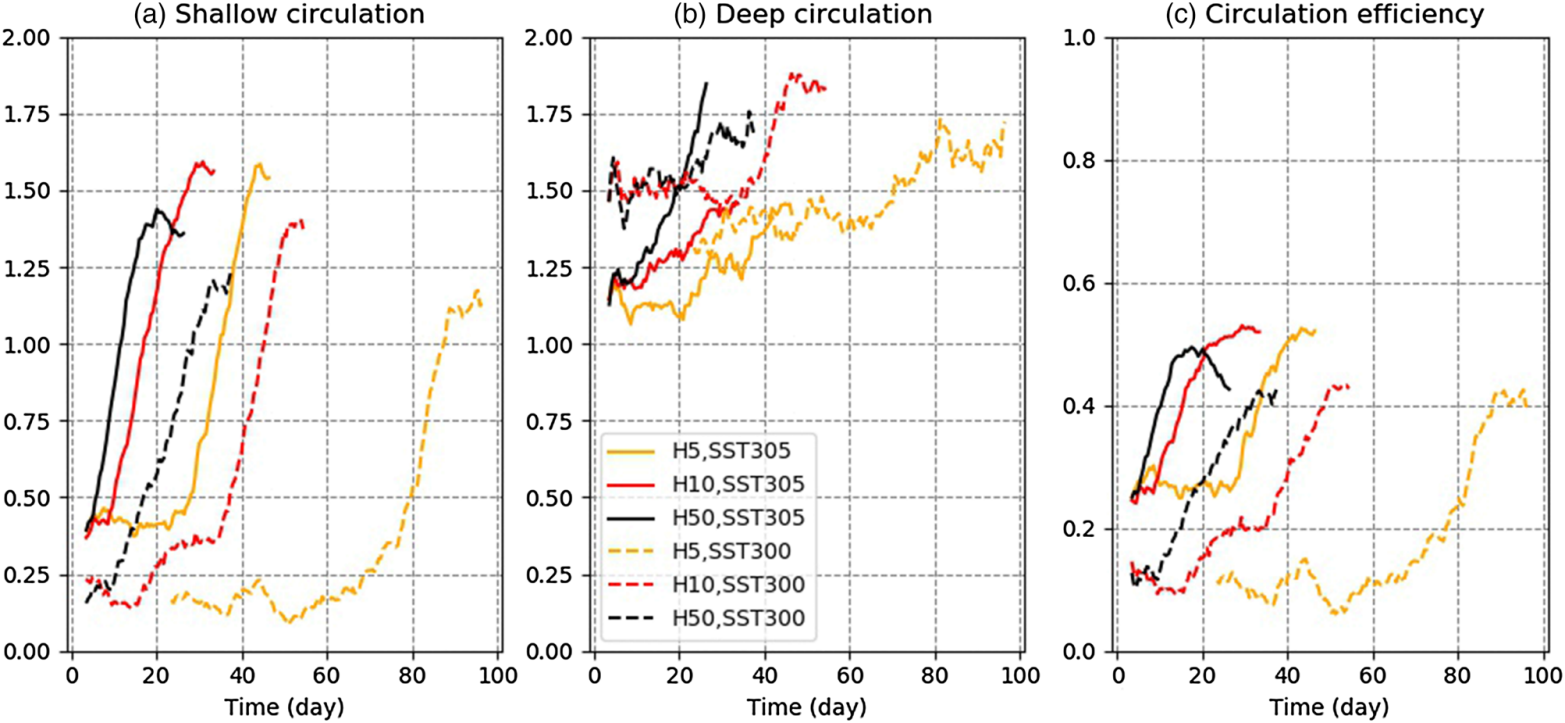
Time evolution of the (a) shallow, (b) deep circulation strength, and (c) the circulation efficiency *η*. Plots are smoothed using a running mean with a 7 day window.

In summary, the longer latency at shallower slabs can be understood by the warmer SST anomaly in dry regions (Figure 8b), leading to reduced surface pressure and thus reduced boundary layer divergence (Figure  9a). But the SST anomaly is very similar at cold (300) and warm (305) SST, at least at early times. Thus, the longer latency at colder SSTs is instead due to weaker boundary layer cooling (Figure 8a), reducing the radiatively driven divergence.

#### 4.1.2 Transient

During the transient period, the aggregation index (Figure 1) monotonically increases to reach its maximum. As we will see here, the slope of the index evolution, which determines the timing of the aggregation, depends on the strength of the shallow circulation and associated up‐gradient transport of MSE, itself function of *Q*_*BL*_ and *SST* anomalies.

Figure 8 shows that, after the latency period, both stronger radiative cooling and colder SST anomalies contribute to the high surface pressure in dry regions. The largest change comes from the SST anomaly, which drops sharply in the H10 and H5 simulations (Figure 8b); the increase in radiative cooling is more gradual and stronger in the 305 K simulations. Once these two cooling effects both contribute to higher surface pressure, aggregation progresses faster, consistent with the increase in the aggregation index (Figure 1).

Thus, with interactive SST, the SST anomalies have an opposition—acceleration impact on the shallow circulation. The early warm anomaly in dry regions opposes the radiatively driven divergent flow and concomitant export of MSE, thus opposing the aggregation, but the later cold anomaly in dry regions reinforces the divergent flow, thus reinforcing the aggregation.

This is well captured in Figure 9a, which shows the strength of the shallow circulation (Ψ_*max*_ − Ψ_*min*_; see section 3.4) in the various simulations. For the shallower slabs, the shallow circulation has a fairly constant value at the beginning (latency regime) followed by a rapid monotonic increase, while for deeper slabs, the SST anomalies are very small, so the “opposition‐acceleration” impact is absent and the shallow circulation has a monotonic increase from the beginning. The deep circulation, on the other hand (Figure 9b), does not show a strong dependence on slab depth or on SST.

These results suggest that the aggregation speed is determined by the shallow circulation between dry and moist regions. Figure 9b also suggests that the deep circulation is not directly linked to the aggregation speed, though we note that the free‐tropospheric drying (Figure 3), which strongly affects the boundary layer cooling and thus the shallow circulation, is closely related to the deep circulation. Although not directly linked to the dry region strengthening and expansion, the deep circulation could therefore play a role in the onset of aggregation, through its impact on boundary layer radiation. We explore this in more detail in the next section.

### 4.2 Link With the Strength of the Shallow Circulation Compared to the Deep Circulation

The vertical structure of the stream function (Figure 7) shows two cells: a shallow circulation with a maximum below 3 km or so and a deeper cell with a maximum around 10 km or so. As noted earlier section 3.4, the shallow circulation is given by Ψ_*max*_ − Ψ_*min*_ and is the fraction of the boundary layer divergence out of dry columns to moist columns, which returns back to dry columns below 4 km or so.

To capture more accurately the shallow circulation, and its relative contribution to the total circulation (shallow plus deep, where the deep circulation as noted in section 3.4, is given by Ψ_*max*,*deep*_), we introduce a circulation efficiency *η*:

(9) $$\eta =\frac{\Psi_{max}-\Psi_{min}}{\Psi_{max,deep}+\Psi_{max}-\Psi_{min}}.$$

The numerator is the fraction of boundary layer divergence out of dry regions into moist regions, which returns to the dry regions below the height of the minimum, 4 km or so. It thus indeed quantifies the shallow circulation. The denominator is the sum of this shallow circulation and of the deep circulation. The latter includes air that has converged into moist regions at low levels as well as in the free troposphere (between the minimum and the deep maximum) and returns to the dry regions above the height of the deep maximum, above 10 km or so. The denominator thus quantifies the overall large‐scale circulation, measured by the total mass transport between dry and moist regions. So *η* (between 0 and 1) measures the fraction of mass transport between dry and moist regions, which is done by the shallow circulation.

Figure 9c shows the time evolution of the circulation efficiency in the various simulations. The link with the aggregation evolution is clear: The simulations with higher circulation efficiency have faster aggregation (Figure 1). This is consistent with earlier studies of self‐aggregation, which highlight the key role played by the MSE transport of the shallow circulation. From the previous section, the shallow circulation is driven by boundary layer radiative cooling and SST anomalies in dry regions, generating hydrostatic high surface pressure anomalies through cooling and virtual effects.

As noted earlier, Figure 9b suggests little contribution from the deep circulation. But larger *Q*_*BL*_ can occur in response to relatively drier upper free troposphere, itself connected to the efficiency of the deep circulation via subsidence drying. Thus, although the deep circulation has little contribution to the aggregation progress or dry region strengthening, we cannot rule out its contribution to the onset of aggregation and of dry regions. The subsidence drying in the free troposphere, which can be seen in Figure 3, could play an important role in initiating the boundary layer cooling enhancement in dry regions, which then amplifies the drying, high pressure, and low‐level divergence.

**Figure 10:**
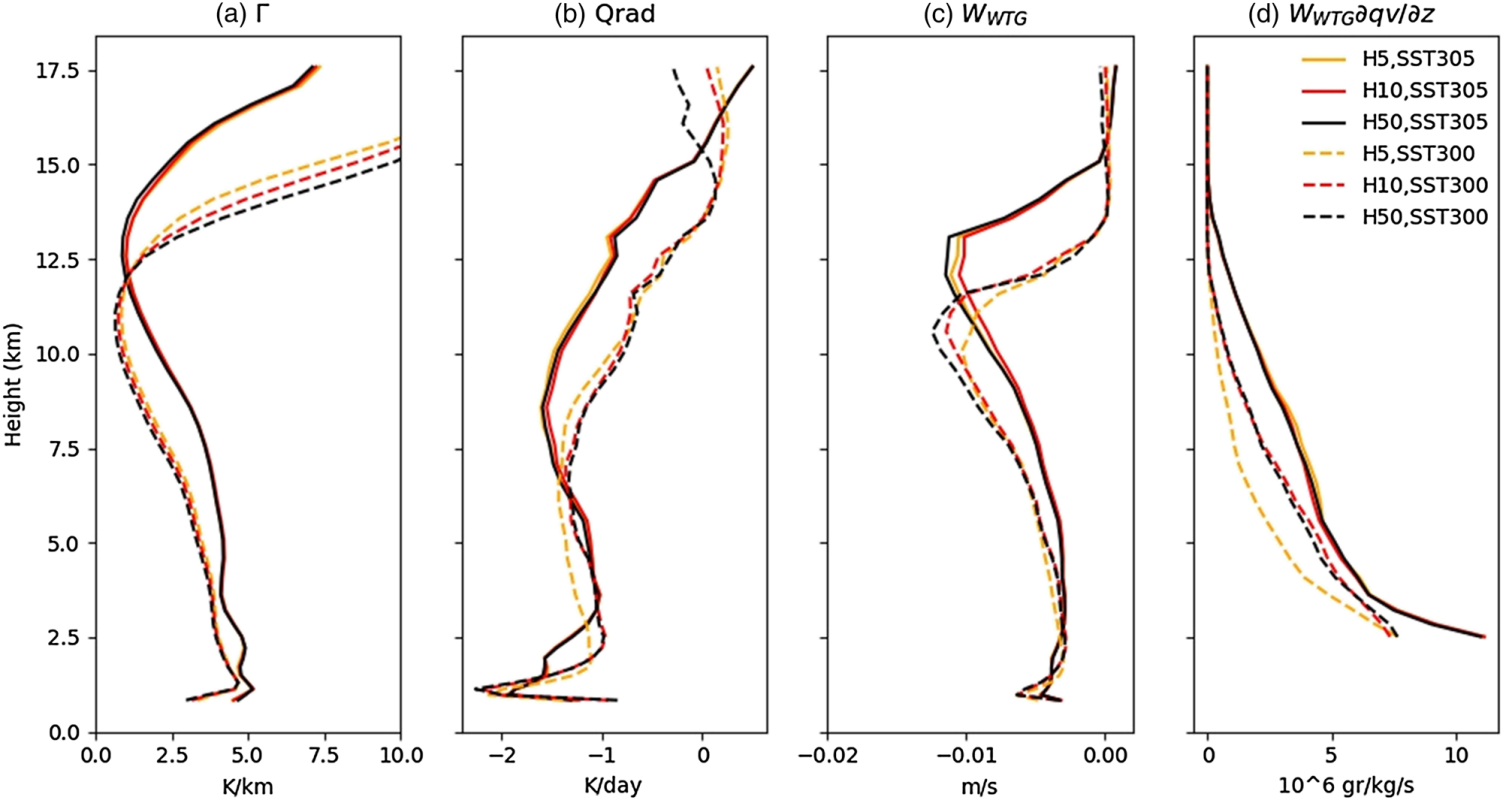
Plot shows (a) the stratification, (b) atmospheric radiative cooling, (c) vertical velocity computed by WTG, and (c) dryness tendency. The variables are averaged over transient part (0.2 < *δCRH* < 0.5 in Figure 1).

The deep circulation is determined by the upward mass transport in deep moist convection, and in our doubly periodic domain by the compensating subsidence in cloud‐free areas. The strength of the subsidence velocity in cloud‐free areas can be quantified using the weak temperature gradient (WTG) approximation as follows:

(10) $$w_{WTG}=\frac{Qrad}{\Gamma },$$

where

(11) $$\Gamma =\frac{T}{\theta }\frac{d\theta }{dz}$$

is the stratification, *Qrad* is the radiative cooling, *T* is temperature, and *θ* is potential temperature. Our findings show that (Figure 10) *Qrad* in the dry region is larger at higher SST (Figure 10b) but so is Γ (Figure  10a) as has been mentioned by Jenney et al. (2020), so that *W*_*WTG*_ does not explain the different timings of aggregation between the two SSTs (Figure 10c): for instance, H5SST305 and H5SST300 have approximately the same maximum *W*_*WTG*_ while their aggregation speed is very different (Figure 1); we note in passing that the maximum *W* has similar magnitude but occurs higher in the warmer simulation, consistent with theoretical expectations (Singh & O'Gorman, 2012).

But enhanced drying is still expected from subsidence, as it is given by *W*_*WTG*_*∂q*_*v*_/*∂z*, and the gradient of specific humidity *∂q*_*v*_/*∂z* is larger at warmer SSTs (Figure 10d). It should be mentioned that the horizontal tendency is similar between the simulations with different SSTs and slab depths (not shown) so that, to the first order, the total tendency is dictated by the vertical term, *W*_*WTG*_*∂q*_*v*_/*∂z*. In the subsiding regions, the free tropospheric drying is thus expected to be stronger at warmer SST, enhancing the boundary layer radiative cooling and the radiatively driven divergence. We suggest that the positive PSFC anomaly in the dry region is the organizer of the convective clouds by exporting low‐level moist air from the dry patches and expanding them. But the formation of a positive PSFC anomaly is a response to the large‐scale deep circulation and free‐tropospheric drying and is necessary for the persistence and expansion of dry patches leading to the confinement of convection.

## 5 Conclusions

The importance of SST on the aggregation of convective clouds has been shown in earlier studies, however, mostly using a constant and uniform SST (e.g., Wing, 2019). Here we study the feedbacks between an interactive SST and the aggregation of convective clouds using a 3‐D RCE setup. To have an interactive surface temperature, we use a slab ocean with fixed mean SST but locally varying temperature.

Consistent with earlier studies, we find that the presence of an interactive SST delays the aggregation of convection, and the stronger the interaction (smaller slab depth), the longer the delay. It has been suggested (Hohenegger & Stevens, 2016) that this delay could be due to the development of an SST gradient between dry and moist regions, with a warm anomaly in dry regions opposing the shallow circulation believed to play a crucial role in the aggregation process. Indeed, the onset of aggregation, and the associated strengthening and expansion of dry regions, is associated with a shallow circulation (below 4 km or so). This shallow circulation diverges from dry regions near the surface and in the boundary layer and returns back into dry regions just above the boundary layer. It thus transport MSE from dry into moist regions, that is, up‐gradient, and has negative gross moist stability, reinforcing MSE gradients and leading to aggregation (Bretherton et al., 2005; Muller & Held, 2012).

We find that the surface temperature anomaly underneath the dry patches actually depends on their dryness. At first, the dryness is small, and warming by shortwave radiation dominates the cooling by latent heat flux and surface longwave radiation, so that the surface under the dry patch warms. But when the dryness becomes large, the surface can cool more efficiently due to predominantly enhanced surface longwave radiative cooling, as the column is dry and downward longwave radiation reduces (the dryness also results in the enhancement of LHF though it is small compared to longwave radiative cooling).

This first positive and then negative SST anomaly in dry regions has an impact on the surface pressure anomaly in dry regions, which is important for driving the aforementioned shallow circulation and guaranteeing the growth and expansion of the dry patch. When positive, the SST anomaly under the dry patch opposes the divergent boundary layer flow out of the dry patch, while if negative, it adds to the positive PSFC anomaly and further helps the divergent flow and concomitant expansion of the dry patch. However, even with an initially positive SST anomaly, the dry patch has a positive PSFC, due to enhanced boundary layer radiative cooling. This radiative cooling decreases the boundary layer temperature and humidity via subsidence drying, resulting in higher PSFC (by virtual effects). Then, when the SST anomaly becomes negative, it adds to the high PSFC anomaly and thus enhances the divergent flow from dry regions and the aggregation. So the SST anomaly has an opposition—acceleration impact on aggregation—and the differential boundary layer radiative cooling and virtual effects are necessary for triggering the aggregation.

In the Earth's tropics, the surface temperature in clear‐sky areas is usually warmer than the surrounding as a result of enhanced shortwave radiation at the surface, broadly corresponding to the initial warming discussed above. The second cooling phase discussed above, which occurs after tens of days, requires a dry atmosphere. In our simulation at 300 K, when the precipitable water at the center of the dry patch reaches below about 15 mm (corresponds to CRH ≈0.4), the net surface radiation can have a cooling effect. Using ERA5 reanalysis, we find that over the tropics, PW ≤15 mm is not rare. However, the cooling effect is a slow process, and its time scale depends on the depth of ocean mixed layer. In our CRM simulations with double periodicity, the dry patches are persistent enough for the surface cooling by radiation to become important. Whether this can be the case over the tropics deserves further investigation.

We note that although the PSFC anomalies originate from boundary layer virtual temperature anomalies, they are the result of the response of the boundary layer to the free tropospheric moisture reduction. It is indeed the free‐tropospheric drying, which allows larger boundary layer radiative cooling and injection of free tropospheric dry air into the boundary layer. The PSFC anomaly is thus the organizer of convection, or the amplifier of aggregation, by creating the shallow circulation which transports low‐level air with high MSE up‐gradient and favors aggregation. But the free‐tropospheric drying appears to be initiating the process. A circulation efficiency, which measures the strength of the shallow circulation relative to the overall circulation (shallow + deep), is found to correlate well with the speed of aggregation. Interestingly, the shallow circulation strength increases strongly in the simulations as aggregation proceeds, while the deep circulation remains much more constant. When the SST anomaly in the dry patch turns negative, the shallow circulation shows a clear strengthening due to the increased high PSFC anomaly.

Using different slab depths and SSTs, our findings confirm that the aggregation is faster with deeper slab and at higher SST. When the slab is shallow, warm SST anomalies that form at the early stage of dry patches weakens the pressure anomalies thus the shallow circulation. This weakening delays the aggregation. We also find that at higher SST, the boundary layer radiative cooling is larger for the same dryness, so that the negative impact of the warm SST anomaly on PSFC is less important compared to the positive impact of the stronger boundary layer radiative cooling.

The initial free‐tropospheric drying is also found to be sensitive to SST. The free tropospheric radiative cooling is stronger at higher SST (but largely independent of slab depth for a given SST). However, the atmospheric stratification is also larger at higher SST so that the free‐tropospheric subsidence velocity calculated using the WTG approximation (Sobel et al., 2001) does not explain the different speeds of aggregation in our simulations. But the resulting free‐tropospheric moisture tendency is larger at higher SST. Larger free‐tropospheric drying at warmer SST yields enhanced boundary layer radiative cooling, itself leading to the formation of positive PSFC anomalies. Therefore, the boundary layer shallow circulation determines the speed of aggregation, but the deep circulation and the associated free‐tropospheric drying appear to be necessary for the formation of a high surface pressure anomaly in dry regions leading to the shallow circulation.

In this study we focused on the role of the shallow circulation (which we defined as the part of the shallow flux from dry to moist regions that stays in the boundary layer, circulation below 4 km or so) and associated MSE transport. However, it is important to mention that the total shallow flux (the maximum of stream function in the boundary layer, which also includes the part of the boundary layer divergence that returns to the dry region higher in altitude) shows the same pattern as shallow circulation: It increases with aggregation, and it is strongly correlated with aggregation index (Figure S1 in the supporting information). Whether shallow circulation is important for aggregation or shallow flux would be an interesting question to further investigate. The main difference is that the former one is more correlated with MSE transport while the latter one with moisture transport. Which one, if any, dominates the aggregation process and concomitant strengthening of energy and moisture gradients is unclear.

## Supporting information

Figure S1

## Figures and Tables

**Figure A1:**
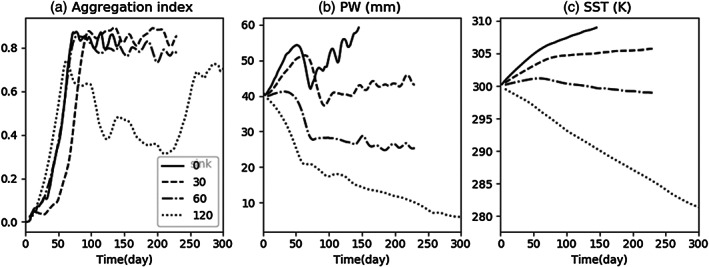
Time evolution of (a) aggregation index, (b) PW, and (c) domain mean surface temperature for simulations with constant sink equal to 0 W m^−2^ (solid line), 30 W m^−2^ (dashed line), 60 W m^−2^ (dashed dotted line), and 120 W m^−2^ (dotted line).

**Figure B1:**
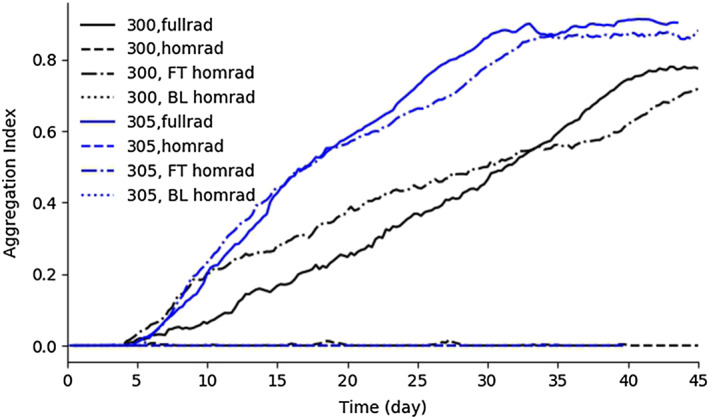
Plot shows the aggregation index time series for the sensitivity tests at 300 K (black curves) and 305 K (blue curves). Homrad, FT, and BL stand for homogenized radiation, free troposphere, and boundary layer, respectively.

## Data Availability

Data used in this study will be available online (at https://doi.org/10.6084/m9.figshare.12249743.v1).

## A Impact of Interactive SST With Constant Deep Ocean Sink

### A.1

As mentioned in section 2.3, the incoming solar flux in the tropics exceeds the threshold for runaway greenhouse warming (Pierrehumbert, 2010). As a consequence, in closed domain simulations with interactive SST such as those used here, without the oceanic and atmospheric transport of energy out of the tropics, the SST increases without bound (Hohenegger & Stevens, 2016). Relaxing the domain mean SST to a target SST, as is done in this study, allows to prevent this runaway greenhouse warming and maintains the domain mean SST close to constant.

Another possibility to avoid the runaway greenhouse warming is to add a sink of energy, for example, into the deep ocean (following Romps, 2011). Figure A1 shows the time evolution of the aggregation index, domain‐mean PW, and domain‐mean SST for simulations where a spatially and temporally constant ocean sink of energy of varying strength (from 0 to 120 W m^−2^) is added to the surface energy budget (negative term added to the right‐hand side of Equation 2 instead of the relaxation term on the left‐hand side). The slab depth is 10 m, and the initial temperature is 300 K for all simulations. In order to investigate the sensitivity to the amount of sink, four different sinks of energy are tested: 0, 30, 60, and 120 W m^−2^.

For all four sinks, the aggregation proceeds. For sink = 0 W m^−2^, as expected, the domain‐mean SST increases quickly. The aggregation reduces the increasing trend of SST (as aggregation is associated with increased Outgoing Longwave Radiation, or OLR, cooling, e.g., Wing & Emanuel, 2014) but does not stop it, as the amount of energy reaching the surface is still large and it is thus not in equilibrium energetically. PW shows a sharp reduction with aggregation, but with surface warming, it continues increasing. For sink = 30 W m^−2^, the domain mean SST has a very modest increase after aggregation as the sink of energy is very close to the surface energy imbalance when aggregated (40 W m^−2^). The simulation with sink = 60 W m^−2^ starts cooling after aggregation but does not disaggregate with cooling (not shown) as the aggregation is favored even at cold *SST* = 295 K in our simulations (Shamekh et al., 2019).

When the energy sink is large, equal to 120 W m^−2^, the surface cools strongly, and the cooling is enhanced by aggregation. Interestingly, this simulation disaggregates partially around *SST* = 295 K and Day 50, but aggregates again when the SST drops below 285 K around Day 220. The disaggregation‐reaggregation can be seen easily in Figure A1a.

It has been as was hypothesized in past studies (e.g., Khairoutdinov & Emanuel, 2010) that self‐aggregation could be a way for tropical temperatures to regulate themselves. Indeed, since aggregation is associated with increased OLR, it was suggested that as SST increases, self‐aggregation is favored, yielding more OLR cooling and reducing the SST back toward its initial value. So maybe the tropical atmosphere could “regulate” its temperature through modulation of its convective aggregation. However, the oscillation between aggregation‐disaggregation does not happen in our simulations (or it is very sensitive to the ocean sink), as the aggregation is favored over a large range of SST.

In summary, independent of the sink, all the simulations aggregate. But the final SST and PW of the simulations depend on the sink of energy. In order to minimize drift in domain mean SST and also to avoid the sensitivity of results to the strength of the imposed ocean sink, in this study we instead avoid the runaway greenhouse climate by relaxing the domain mean SST toward a target temperature.

## B Sensitivity Simulations and Low‐Level Radiative Cooling

### B.1

In order to verify the importance of boundary layer radiative cooling, we run three sensitivity tests by homogenizing the radiative cooling profile (1) at all levels (mentioned as homrad in Figure B1), (2) in the boundary layer, which we crudely define to be up to 1 km above the surface (referred to as BL‐homorad), and (3) in free troposphere, above 1 km to the top of the domain (mentioned as FT‐homrad).

As Figure B1 shows for both temperatures, 300 and 305 K, radiative cooling feedbacks are necessary for self‐aggregation so that homogenizing the radiation profile prevents the self‐aggregation. The results also show that for FT‐ homrad, in which the radiation is homogenized only in free troposphere, the aggregation proceeds without significant difference from the control simulations (fullrad). But homogenizing the boundary layer radiative feedback (BL‐homrad in Figure B1) prevents the aggregation. These findings are consistent with Bretherton et al. (2005), who emphasize the role of low‐tropospheric/boundary layer radiative cooling, as well as with Muller and Held (2012), who find the shallow clouds radiative feedbacks to be important for the aggregation onset.

## References

[jame21206-bib-0001] Arnold, N. P. , & Randall, D. A. (2015). Global‐scale convective aggregation: Implications for the Madden‐Julian Oscillation. Journal of Advances in Modeling Earth Systems, 7, 1499–1518. 10.1002/2015MS000498

[jame21206-bib-0002] Back, L. E. , & Bretherton, C. S. (2009). On the relationship between SST gradients, boundary layer winds, and convergence over the tropical oceans. Journal of Climate, 22(15), 4182–4196. 10.1175/2009JCLI2392.1

[jame21206-bib-0003] Bretherton, C. S. , Blossey, P. N. , & Khairoutdinov, M. (2005). An energy‐balance analysis of deep convective self‐aggregation above uniform SST. Journal of the Atmospheric Sciences, 62(12), 4273–4292. 10.1175/JAS3614.1

[jame21206-bib-0004] Collins, W. D. , Rasch, P. J. , Boville, B. A. , Hack, J. J. , McCaa, J. R. , Williamson, D. L. , Briegleb, B. P. , Bitz, C. M. , Lin, S. J. , & Zhang, M. (2006). The formulation and atmospheric simulation of the Community Atmosphere Model Version 3 (CAM3). Journal of Climate, 19(11), 2144–2161. 10.1175/JCLI3760.1

[jame21206-bib-0005] Coppin, D. , & Bony, S. (2015). Physical mechanisms controlling the initiation of convective self‐aggregation in a general circulation model. Journal of Advances in Modelling Earth Systems, 7, 2060–2078. 10.1002/2015MS000571

[jame21206-bib-0006] Coppin, D. , & Bony, S. (2017). Internal variability in a coupled general circulation model in radiative‐convective equilibrium. Geophysical Research Letters, 44, 5142–5149. 10.1002/2017GL073658

[jame21206-bib-0007] Coppin, D. , & Bony, S. (2018). On the interplay between convective aggregation, surface temperature gradients, and climate sensitivity. Journal of Advances in Modeling Earth Systems, 10, 3123–3138. 10.1029/2018MS001406 31007836 PMC6472628

[jame21206-bib-0008] Craig, G. C. , & Mack, J. M. (2013). A coarsening model for self‐organization of tropical convection. Journal of Geophysical Research: Atmospheres, 118, 8761–8769. 10.1002/jgrd.50674

[jame21206-bib-0009] Cronin, T. W. , Emanuel, K. A. , & Molnar, P. (2015). Island precipitation enhancement and the diurnal cycle in radiative‐convective equilibrium. Quarterly Journal of the Royal Meteorological Society, 141(689), 1017–1034.

[jame21206-bib-0010] Emanuel, K. A. , Wing, A. A. , & Vincent, E. M. (2014). Radiative‐convective instability. Journal of Advances in Modelling Earth Systems, 6, 75–90. 10.1002/2013MS000270

[jame21206-bib-0011] Grabowski, W. W. (2006). Impact of explicit atmosphere ‐ ocean coupling on MJO‐like coherent structures in idealized aquaplanet simulations. Journal of the Atmospheric Sciences, 63(9), 2289–2306. 10.1175/JAS3740.1

[jame21206-bib-0012] Held, I. M. , Hemler, R. S. , & Ramaswamy, V. (1993). Radiative‐convective equilibrium with explicit two‐dimensional moist convection. Journal of the Atmospheric Sciences, 50(23), 3909–3909.

[jame21206-bib-0013] Hohenegger, C. , & Stevens, B. (2016). Coupled radiative convective equilibrium simulations with explicit and parameterized convection. Journal of Advances in Modelling Earth Systems, 8, 1468–1482. 10.1002/2016MS000666

[jame21206-bib-0014] Jeevanjee, N. , & Romps, D. M. (2013). Convective self‐aggregation, cold pools, and domain size. Geophysical Research Letters, 40, 994–998. 10.1002/grl.50204

[jame21206-bib-0015] Jenney, A. M. , Randall, D. A. , & Branson, M. D. (2020). Understanding the response of tropical ascent to warming using an energy balance framework. Journal of Advances in Modeling Earth Systems, 12, e2020MS002056 10.1029/2020MS002056

[jame21206-bib-0016] Khairoutdinov, M. F. , & Emanuel, K. A. (2010). Aggregated convection and the regulation of tropical climate. In *Preprints, 29th conference on Hurricanes and Tropical Meteorology, Tucson, AZ, Amer. Meteor. Soc.P2.69*.

[jame21206-bib-0017] Khairoutdinov, M. F. , & Randall, D. A. (2003). Cloud resolving modeling of the ARM summer 1997 IOP: Model formulation, results, uncertainties, and sensitivities. Journal of the Atmospheric Sciences, 60(4), 607–625. 10.1175/1520-0469(2003)060<0607:CRMOTA>2.0.CO;2

[jame21206-bib-0018] Liu, C. , & Moncrieff, M. W. (2008). Explicitly simulated tropical convection over idealized warm pools. Journal of Geophysical Research, 113, D21121 10.1029/2008JD010206

[jame21206-bib-0019] Mauritsen, T. , & Stevens, B. (2015). Missing iris effect as a possible cause of muted hydrological change and high climate sensitivity in models. Nature Geoscience, 8(5), 346–351. 10.1038/ngeo2414

[jame21206-bib-0020] Muller, C. J. , & Bony, S. (2015). What favors convective aggregation and why? Geophysical Research Letters, 42, 5626–5634. 10.1002/2015GL064260

[jame21206-bib-0021] Muller, C. J. , & Held, I. M. (2012). Detailed investigation of the self‐aggregation of convection in cloud‐resolving simulations. Journal of the Atmospheric Sciences, 69, 2551–2565. 10.1175/JAS-D-11-0257.1

[jame21206-bib-0022] Müller, S. K. , & Hohenegger, C. (2020). Self‐Aggregation of convection in spatially varying sea surface temperatures. Journal of Advances in Modeling Earth Systems, 12, e2019MS001698 10.1029/2019MS001698 PMC7685139 33282117

[jame21206-bib-0023] Nakajima, K. , & Matsuno, T. (1988). Numerical experiments concerning the origin of cloud clusters in the tropical atmosphere. Journal of the Meteorological Society of Japan. Ser. II, 66(2), 309–329. 10.2151/jmsj1965.66.2_309

[jame21206-bib-0024] Naumann, A. K. , Stevens, B. , & Hohenegger, C. (2019). A moist conceptual model for the boundary layer structure and radiatively driven shallow circulations in the trades. Journal of the Atmospheric Sciences, 76(5), 1289–1306.

[jame21206-bib-0025] Naumann, A. K. , Stevens, B. , Hohenegger, C. , & Mellado, J. P. (2017). A conceptual model of a shallow circulation induced by prescribed low‐level radiative cooling. Journal of the Atmospheric Sciences, 74(10), 3129–3144.

[jame21206-bib-0026] Neelin, J. D. , & Held, I. M. (1987). Modeling tropical convergence based on the moist static energy budget. Monthly Weather Review, 115(1), 3–12.

[jame21206-bib-0027] Pierrehumbert, R. T. (2010). Principles of planetary climate. Cambridge, UK: Cambridge University Press.

[jame21206-bib-0028] Raymond, D. J. , Sessions, S. L. , Sobel, A. H. , & Fuchs, Z. (2009). The mechanics of gross moist stability. Journal of Advances in Modelling Earth Systems, 1, 9 10.3894/JAMES.2009.1.9

[jame21206-bib-0029] Romps, D. M. (2011). Response of tropical precipitation to global warming. Journal of the Atmospheric Sciences, 68(1), 123–138.

[jame21206-bib-0030] Semie, A. G. , & Tompkins, A. M. (2016). Organization of tropical convection in low vertical wind shears: Impact of boundary conditions. In *EGU General Assembly Conference Abstracts* (Vol. 18), Vienna, Austria.

[jame21206-bib-0031] Shamekh, S. , Muller, C. , Duvel, J.‐P. , & D'Andrea, F. (2019). How do ocean warm anomalies favor the aggregation of deep convective clouds? Journal of the Atmospheric Sciences. 10.1175/JAS-D-18-0369.1

[jame21206-bib-0032] Singh, M. S. , & O'Gorman, P. A. (2012). Upward shift of the atmospheric general circulation under global warming: Theory and simulations. Journal of Climate, 25, 8259–8276.

[jame21206-bib-0033] Sobel, A. H. , Nilsson, J. , & Polvani, L. M. (2001). The weak temperature gradient approximation and balanced tropical moisture waves. Journal of the Atmospheric Sciences, 58, 23 10.1175/1520-0469(2001)058<3650:TWTGAA>2.0.CO;2

[jame21206-bib-0034] Tompkins, A. M. (2001). Organization of tropical convection in low vertical wind shears: The role of water vapor. Journal of the Atmospheric Sciences, 58(6), 529–545. 10.1175/1520-0469(2001)058<0529:OOTCIL>2.0.CO;2

[jame21206-bib-0035] Tompkins, A. M. , & Craig, G. C. (1998). Radiative‐convective equilibrium in a three‐dimensional cloud‐ensemble model. Quarterly Journal of the Royal Meteorological Society, 124, 2073–2097. 10.1002/qj.49712455013

[jame21206-bib-0036] Wing, A. (2019). Self‐aggregation of deep convection and its implications for climate. Current Climate Change Reports, 5(1), 1–11.

[jame21206-bib-0037] Wing, A. , & Cronin, T. W. (2016). Self‐aggregation of convection in long channel geometry. Quarterly Journal of the Royal Meteorological Society, 142(694), 1–15. 10.1002/qj.2628

[jame21206-bib-0038] Wing, A. , & Emanuel, K. (2014). Physical mechanisms controlling self‐aggregation of convection in idealized numerical modeling simulations. Journal of Advances in Modelling Earth Systems, 6, 59–74. 10.1002/2013MS000269

[jame21206-bib-0039] Wing, A. , Emanuel, K. , Holloway, C. E. , & Muller, C. J. (2017). Convective Self‐Aggregation in numerical simulations: A review. Surveys in Geophysics, 38(6), 1173–1197. 10.1007/978-3-319-77273-8_1

[jame21206-bib-0040] Yang, D. (2018). Boundary layer diabatic processes, the virtual effect, and convective self‐aggregation. Journal of Advances in Modeling Earth Systems, 10, 2163–2176. 10.1029/2017MS001261

