# Supplementary figures and images for: Self‐Aggregation of Convective Clouds With Interactive Sea Surface Temperature
Source: J Adv Model Earth Syst. 2020 Nov 3;12(11):e2020MS002164. doi: 10.1029/2020MS002164 (PMC7685139; doi:10.1029/2020MS002164)

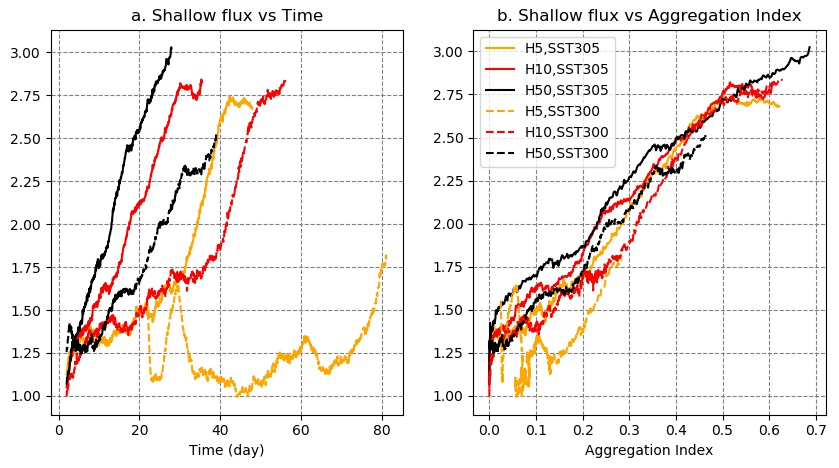

Supplement: Supplementary file 1 — Figure S1 [file JAME-12-e2020MS002164-s001.jpg]
